# Dynamic Intelligent Scheduling in Low-Carbon Heterogeneous Distributed Flexible Job Shops with Job Insertions and Transfers

**DOI:** 10.3390/s24072251

**Published:** 2024-03-31

**Authors:** Yi Chen, Xiaojuan Liao, Guangzhu Chen, Yingjie Hou

**Affiliations:** 1 College of Computer Science and Cyber Security, Chengdu University of Technology, Chengdu 610059, China; chenyee@stu.cdut.edu.cn (Y.C.); chenguangzhu2012@cdut.edu.cn (G.C.); 2021050834@stu.cdut.edu.cn (Y.H.); 2 Sichuan Engineering Technology Research Center for Industrial Internet Intelligent Monitoring and Application, Chengdu 610059, China

**Keywords:** heterogeneous distributed flexible job shop, dynamic scheduling, low-carbon, deep reinforcement learning, Rainbow DQN

## Abstract

With the rapid development of economic globalization and green manufacturing, traditional flexible job shop scheduling has evolved into the low-carbon heterogeneous distributed flexible job shop scheduling problem (LHDFJSP). Additionally, modern smart manufacturing processes encounter complex and diverse contingencies, necessitating the ability to address dynamic events in real-world production activities. To date, there are limited studies that comprehensively address the intricate factors associated with the LHDFJSP, including workshop heterogeneity, job insertions and transfers, and considerations of low-carbon objectives. This paper establishes a multi-objective mathematical model with the goal of minimizing the total weighted tardiness and total energy consumption. To effectively solve this problem, diverse composite scheduling rules are formulated, alongside the application of a deep reinforcement learning (DRL) framework, i.e., Rainbow deep-Q network (Rainbow DQN), to learn the optimal scheduling strategy at each decision point in a dynamic environment. To verify the effectiveness of the proposed method, this paper extends the standard dataset to adapt to the LHDFJSP. Evaluation results confirm the generalization and robustness of the presented Rainbow DQN-based method.

## 1. Introduction

In recent years, due to the progress of globalization and computer technology, numerous manufacturing enterprises have shifted from the traditional single job shop model to the distributed job shop model. This shift can reduce labor and raw material costs while improving production efficiency. In contrast to the classical flexible job shop scheduling problem (FJSP), the heterogeneous distributed flexible job shop scheduling problem (HDFJSP) surpasses the constraints on the uniqueness of job shops. Each job can be dispatched to multiple job shops in various locations, and each operation can be allocated to more than one candidate machine. As a result, the distributed production mode is more flexible and better suited for the actual production environment.

Furthermore, industry is the world’s second-largest source of carbon dioxide emissions, with total emissions of approximately 870 million tons of carbon dioxide in 2020, and the energy consumption of the manufacturing industry is expected to rise to 16 percent in 2030 [1]. In China, the manufacturing sector accounted for roughly 84 percent of total industrial energy consumption in 2020, with electricity consumption in the sector increasing by 3 percent from 2019, as reported by the U.S. Energy Information Administration [2]. Meanwhile, in the United States, the industrial sector consumes approximately 33.3 percent of energy from various sources, including fossil fuels, renewable energies, and nuclear power, according to the U.S. Energy Information Administration [3]. In the Industry 4.0 era, due to increasing energy costs and environmental pollution, the low-carbon scheduling problem has garnered significant attention from academics and engineers as a new mode of dispatch.

Moreover, the practical production environment encounters more complex and diverse contingencies. In the event of an emergency, rescheduling from scratch is not only time-consuming, but also demands significant expert experience. As a result, it becomes challenging to meet the requirements of a real-time production environment while maintaining superior scheduling quality [4,5].

In summary, the dynamic multi-objective scheduling problem (DMoSP) for low-carbon heterogeneous distributed flexible job shop (LHDFJS) is a novel and significant topic that is relevant to modern supply chain and manufacturing systems. The LHDFJS model represents a multi-factory low-carbon production environment where each factory operates as a low-carbon flexible job shop. Besides that, LHDFJS is characterized by a large-scale, complex, and variable environment with numerous constraints and strong dynamics. These factors can lead to unexpected events such as job insertions and machine faults, which can impact the previously generated scheduling scheme or render it invalid [6]. Notably, job shop scheduling is one of the essential methods to reduce carbon emissions in the manufacturing sector. Traditional job shop scheduling strategies have focused primarily on economic factors such as completion time and machine utilization, while neglecting energy and environmental considerations such as energy consumption during processing and transportation. Therefore, the study of the DMoSP for LHDFJS holds significant theoretical significance and practical value.

To solve the distributed flexible job shop scheduling problem, the majority of existing works restrict that all the operations of a job must be processed in the same job shop [7,8,9,10]. Few works [11,12,13] allow for workpieces to be transferred and processed in different job shops, assuming homogenous job shops with equal transportation times between job shops and machines to simplify the problem. However, the heterogeneity of job shops is an important characteristic of DFJS. In the scheduling of heterogeneous job shops, dynamically balancing economic and environmental objectives becomes a key factor for manufacturing enterprises to enhance competitiveness. With this in mind, this paper leverages a Rainbow deep-Q network (Rainbow DQN), to construct a deep reinforcement learning (DRL) framework to tackle the DMoSP for LHDFJS.

Main contributions are listed as follows:

1. Aiming at the DMoSP for LHDFJS, a mathematical model is established with the objective of minimizing the total weighted tardiness and total energy consumption of the processing process. Heterogeneous job shops with different machine processing capabilities and energy consumption, different transportation times between job shops and machines, and job transfers between job shops are all considered;
2. Seven job selecting rules and six machine assignment rules are designed. By Cartesian quadrature, a total of 42 composite scheduling rules are designed to optimize the total weighted tardiness and total energy consumption in an LHDFJSP;
3. A Rainbow DQN is proposed to address the DMoSP for LHDFJS. State space, action space and reward function are all redesigned. Specifically, 10 state features are extracted to summarize the status of production. Forty-two composite scheduling rules are obtained as the candidate actions. A novel reward function, consisting of immediate and episodic rewards, is designed to balance the economic and environmental indicators;
4. A new dataset is extended from the standard one to adapt to the DMoSP for LHDFJS with job transfers and insertions. This allows for a more realistic representation of the scheduling problem and better evaluation of the algorithms;
5. Based on the newly built dataset, this study compares the Rainbow DQN-based method and dueling double DQN with prioritized experience replay (D3QN with PER), as well as 8 classical heuristic scheduling rules and 42 candidate composite scheduling rules. The results indicate that the proposed composite scheduling rules outperform the classical heuristic scheduling rules, while Rainbow DQN excels over other algorithms in minimizing both total weighted tardiness and total energy consumption.

The remainder of this paper is organized as follows: Section 2 provides an overview of the research and practical applications of the DMoSP for LHDFJS. Section 3 primarily introduces the preliminary of the Rainbow DQN. In Section 4, the mathematical model of the DMoSP for LHDFJS is presented. Section 5 describes the application of the Rainbow DQN algorithm in the DMoSP for LHDFJS. In Section 6, experimental analysis of the proposed algorithm and comparison experiments are presented. Finally, Section 7 concludes this paper.

## 2. Literature Review

This section introduces an overview of the current research status in terms of distributed flexible job shop scheduling, low-carbon scheduling, dynamic scheduling, and DRL-based scheduling methods.

### 2.1. Distributed Flexible Job Shop Scheduling

By effectively coordinating multiple workshops and machines, distributed flexible job shop scheduling (DFJS) enables efficient utilization of resources, idle time minimization, production delay reduction, and the overall productivity enhancement. Therefore, distributed manufacturing is gradually emerging as the main production method [9]. De Giovanni and Pezzella [7] first defined the DFJS problem and proposed an improved genetic algorithm to address the problem for small and medium-sized distributed manufacturing units in a single factory. Zhao et al. [10] elaborated on a mixed-integer linear programming (MILP) model of the distributed assembly no-idle flow-shop scheduling problem without job transfers and proposed a water wave optimization algorithm combined with a three-stage variable neighborhood search to minimize assembly completion time. Du et al. [8] and Zhang et al. [14] considered the constraints of crane transportation conditions in DFJS. The former used an optimization algorithm combining estimation of distribution algorithm and variable neighborhood search, while the latter utilized a Q-learning-based hyper-heuristic evolutionary algorithm. Li et al. [15] proposed an improved gray wolf optimizer to solve the DFJS problem without job transfers.

In the field of distributed job shop scheduling, as indicated by Luo et al. [11], most of the existing research dedicated to the DFJS problem assumes that workpieces are only allowed to be processed within a certain job shop, i.e., all the operations of the same job must be processed in the same factory. However, in actual production activities, job transfer between distributed job shops is a key factor to take advantage of different workshops and improve production efficiency. The study of Meng et al. [12] made the first attempt to solve the DFJS problem with transfers using MILP and constraint programming models. Luo et al. [11] proposed a memetic algorithm combining evolutionary algorithms and local search to tackle the DFJS problem with transfers, assuming that the transfer times of all machines and factories are the same. Sang and Tan [13] combined the improved NSGA-III and local search method to solve the HDFJSP with transfers, taking into account the energy factor of the job shop.

The aforementioned works assume either homogeneous job shops or equal transport times between job shops to simplify the problem description and solution. However, job shops are heterogeneous, and the transport time may vary among different job shops. Moreover, dynamic scheduling is not supported in these works.

### 2.2. Low-Carbon Scheduling

Low-carbon scheduling plays a crucial role in the field of job shop scheduling by addressing environmental concerns and promoting sustainable practices. Dai et al. [16] established an energy-aware mathematical model integrating process planning and scheduling, proposed performance evaluation indexes including energy consumption (i.e., basic power consumption, unload power consumption, and cutting power consumption) and maximum time of scheduling, and developed an improved genetic algorithm to obtain the Pareto optimal solution. Zhang et al. [17] proposed a low-carbon scheduling flexible job shop model that takes into account production factors and environmental effects and designed indexes of processing carbon efficiency, part carbon efficiency, and machine tool carbon efficiency to estimate carbon emissions from parts and machine tools. Jiang and Deng [18] proposed a bi-population-based discrete cat swarm optimization algorithm to solve the low-carbon FJSP. The research mainly studies the energy consumption of processing and idle-load. Yin et al. [19] formulated a low-carbon scheduling model and introduced a multi-objective genetic algorithm to optimize productivity, energy efficiency, and noise reduction. Li et al. [20] proposed a multi-objective low-carbon job-shop scheduling problem with a variable processing speed constraint and developed an improved artificial swarm algorithm to minimize the makespan, machine loading, and total carbon emissions (i.e., processing energy consumption, idle energy consumption, and on/off switching energy consumption).

The main objective of low-carbon scheduling is to improve energy efficiency by strategically optimizing scheduling processes. Current research primarily addresses factors such as processing energy consumption, idle energy consumption, transportation energy consumption, and on/off switching energy consumption. Considering that frequent on/off switching may potentially cause damage to equipment, this paper places emphasis on key environmental factors including processing energy consumption, idle energy consumption, and transportation energy consumption.

### 2.3. Dynamic Scheduling and Deep Reinforcement Learning Methods

The majority of traditional DFJS methods mainly consider static scheduling issues, neglecting the importance of dynamic scheduling [21]. Since static schedules are fixed, assuming that all data are known beforehand, they are relatively easier to plan and manage, especially in stable and predictable environments. By contrast, dynamic scheduling optimizes resource utilization based on real-time demand and availability. It minimizes idle time and maximizes productivity by dynamically allocating resources to the most critical tasks. Shahgholi et al. [22] proposed a heuristic model for a dynamic FJSP with variable processing time and rescheduling based on the idea of the artificial bee colony algorithm. Li et al. [23] designed a rescheduling method based on the Monte Carlo tree search algorithm for a dynamic FJSP considering four dynamic contingencies, which can shorten the response time to dynamic contingencies. Applications in dynamic scheduling problems can be divided into completely reactive, robust, and predictive–reactive methods. In recent years, scholars have mainly studied the schemes and performance of robust and predictive–reactive methods in dynamic scheduling [24,25].

With the ability to learn from experience and make intelligent decisions, deep reinforcement learning (DRL) can optimize scheduling strategies and improve overall performance. It enables the system to adapt to dynamic environments, handle uncertainties, and optimize various objectives simultaneously. Yan et al. [26] achieved efficient dynamic scheduling by combining a double-layer Q-learning algorithm with a digital twin algorithm, which considers both machine and worker resources in FJSP and involves four cases of dynamic interference. On the other hand, Chang et al. [27] proposed a double deep Q-network (DDQN) algorithm framework for dynamic scheduling to solve an FJSP with random job arrival times, where the state space, action space, and reward function of the agent were designed. Yan et al. [28] designed a deep Q-network (DQN) framework-based greedy rate reduction to solve the distributed permutation flow shop scheduling problem with machine maintenance constraints. Zhang et al. [29] presented a DRL framework using the proximal policy optimization algorithm to tackle unforeseen machine failures. Li et al. [25] proposed a hybrid DQN for a dynamic FJSP with insufficient transportation resources. Based on the conjugate DQN of DRL algorithm, Lee and Lee [30] proposed a novel state, action, and reward optimization scheduling strategy to achieve self-learning and self-optimizing semiconductor manufacturing systems.

DRL is a promising approach to production scheduling, especially in the stochastic production environment [31]. However, the field of scheduling based on DRL is still in its infancy. Challenges such as lack of interpretability and difficulties in practical industrial applications make designing scheduling solutions based on DRL challenging. Developing a solution that is both competitive and reliable in production scheduling using existing methods is a challenging task.

### 2.4. Summary

Table 1 summarizes the research elements covered in the relevant literature. It can be observed that, regarding the DFJS problem, most studies have overlooked the transfer factors between job shops and have not considered the heterogeneity of job shops. In addition, existing research only addresses partial production requirements, such as the combination of low-carbon considerations and dynamic scheduling, or considers transfer factors combined with multi-objective problems. This is far from covering the complex factors that exist in actual production.

Furthermore, researchers have proposed diverse solutions based on different types of production environments and requirements. Metaheuristic algorithms remain a common solution in various scenarios. Meanwhile, DRL methods are gradually gaining attention and demonstrating potential in solving dynamic problems and achieve real-time scheduling.

In this paper, complicated factors such as low-carbon considerations, dynamics, transfer, and job shop heterogeneity are taken into account, leading to the design of a dynamic scheduling approach that integrates composite scheduling rules with Rainbow DQN. This method aims to provide a comprehensive and effective solution for addressing the complexities of the LHDFJSP.

## 3. Preliminary

Conventional scheduling methods confront formidable challenges, including intricate problem sets, dynamic and fast-changing environments, and the need for multi-objective optimization. The adoption of Rainbow DQN introduces a pioneering approach to address these challenges. This section delves deep into the theoretical underpinnings of Rainbow DQN. Rainbow DQN stands as a significant milestone in the realm of DRL, presenting a fresh theoretical and technical framework to tackle the intricate scheduling dilemmas found in job shop environments.

### 3.1. Q-Learning and DQN

The Q-learning algorithm was first proposed by Watkins [35] in his doctoral dissertation. Tesauro et al. [36] combined reinforcement learning (RL) with neural networks, which work by working against themselves and learning from the results. The Q-learning algorithm uses the Bellman equation to update the Q-value and store it in a Q-table to estimate the Q-value of the corresponding state-action. The updated formula of Q-value is as follows:

(1) $$Q\left(s_{t},\;a_{t}\right)=Q\left(s_{t},\;a_{t}\right)+\alpha [r_{t}+\gamma maxQ\left(s_{t+1},\;a_{t+1}\right)-Q\left(s_{t},\;a_{t}\right)]$$

Here, α is the learning rate and γ is the discount rate. $s_{t+1}$ is the next state and $a_{t+1}$ represents the action selected in state $s_{t+1}$.

Mnih et al. [37] proposed a Q-learning approach to play an Atari game in conjunction with a deep learning network, which is called a deep Q-network (DQN). The DQN mainly adopts the idea of value function approximation, uses the neural network to approximate the value function, and utilizes the method of target Q network and experience replay to train the network, which improves the training speed and stability of the DQN. The method of calculating the target value in a DQN algorithm is shown in Equation (2):

(2) $$Y_{t}^{DQN}=r_{t+1}+\gamma Q^{\pi }\left(s_{t+1},\;\pi \left(s_{t+1};\theta^{-}\right);\theta^{-}\right)$$

Here, π refers to a certain strategy, and $\pi \left(s_{t+1};\theta^{-}\right)=arg\;max_{a}Q\left(s_{t+1},\;a_{t};\theta^{-}\right)$ is a fixed strategy that leads to limited interactions with the environment. In this way, epsilon greedy is often used to add randomness to exploration. $Q^{\pi }\left(s_{t+1},\;a_{t+1}\right)$ represents the cumulative reward expectation of agent choosing action $a_{t+1}$ under state $s_{t+1}$. The error calculation formula (loss function) between the estimated value and the target value in the current state is as in Equation (3):

(3) $$L\left(\theta \right)=E[(r_{t}+\gamma max_{a_{t+1}\in A}Q\left(s_{t+1},\;a_{t+1};\theta^{-}\right)-Q\left(s_{t},\;a_{t};\theta \right){)}^{2}]$$

### 3.2. Double DQN

Van Hasselt et al. [38] proposed a double DQN method to solve the problem of overestimation of Q-learning by decoupling the choice of action and the calculation of the target Q value. The method of calculating the target value in a double DQN algorithm is shown in Equation (4):

(4) $$Y_{t}^{DoubleDQN}=r_{t+1}+\gamma Q\left[s_{t+1},\;arg\;max_{a}Q\left(s_{t+1},a;\theta \right);\theta^{-}\right]$$

The double DQN algorithm constructs two action value functions. The agent determines the action with the evaluation network and calculates the value of the action with the target network when estimating the reward.

### 3.3. Dueling DQN

Wang et al. [39] improved the network structure of DQNs; the method mainly divides the Q-value function to form a dual network. The dueling DQN proposes two value computation branches, one for predicting state values and the other for predicting state-related action advantage values. The state function is only used to predict whether the state is good or bad, while the action advantage function is only used to predict the importance of each action in that state.

(5) $$Q\left(s_{t},\;a_{t};\theta ,\;\alpha ,\;\beta \right)=V\left(s_{t};\theta ,\;\beta \right)+[A\left(s_{t},\;a_{t};\theta ,\;\alpha \right)-\frac{1}{\left|A\right|}\sum_{a_{t+1}}A\left(s_{t},\;a_{t+1};\theta ,\;\alpha \right)]$$

Equation (5) represents the addition of the state function and the action advantage function, but there might be a non-unique solution. Therefore, the unique action advantage value is obtained by subtracting an average value from the action advantage function. Here, θ denotes shared neural network parameters; α and β, respectively, represent the network parameters of state value function and action advantage function.

### 3.4. Noisy Networks

Fortunato et al. [40] replaced the general neural network with a noisy neural network whose weights and parameters were interfered with by noise functions. The noisy network is re-sampled before each action step and the neural network is updated with noise to improve the action exploration capability of the DRL models. The ordinary linear layer of the neural network is expressed as Equation (6):

(6) y=ωx+b

where x is the input layer, ω is the weight matrix, and b is the deviation. The improved linear layer of noise is defined as Equation (7):

(7) $$y\overset{\mathrm{def}}{=}\left(\mu^{\omega }+\sigma^{\omega }⨀\varepsilon^{\omega }\right)x+\mu^{b}+\sigma^{b}⨀\varepsilon^{b}$$

Here, $\mu^{\omega }$ and $\mu^{b}$ are the mean values required to be obeyed by parameters ω and b, $\sigma^{\omega }$ and $\sigma^{b}$ represent the variance brought by noise, and ε is random noise with the same dimension. The noise weight and noise deviation can be expressed as $\omega =\mu^{\omega }+\sigma^{\omega }⨀\varepsilon^{\omega }$ and $b=\mu^{b}+\sigma^{b}⨀\varepsilon^{b}$, respectively.

### 3.5. Multi-Step Reinforcement Learning (RL)

DRL models typically use a single-step temporal difference (TD) algorithm to judge the value of the target. The TD algorithm inherits the advantages of the dynamic programming and Monte Carlo methods to predict state value and optimal policy [41]. However, a single-step TD algorithm will lead to a large bias in the estimation of the target value during the initial training period. Hence, De Asis et al. [42] demonstrated that immediate rewards can be accurately obtained through interaction with the environment. The idea of adopting multi-step learning is to replace a single-step return with an N-step return, so that the target value at the early stage of training can be estimated more accurately, thus speeding up the training. A multi-step RL concept is adopted in Rainbow DQN to construct the N-step return, and its loss function is as follows:

(8) $$L_{N-step}={\left(\overset{N-1}{\underset{k=0}{\sum }}\gamma^{k}r_{t+k}+\gamma^{N}max_{a_{t+1}}Q\left(s_{t+N},\;a_{t+1};\theta^{-}\right)-Q\left(s_{t},\;a_{t};\theta \right)\right)}^{2}$$

### 3.6. Prioritized Experience Replay (PER)

In a conventional DQN, the experience replay is uniformly sampled from the entire experience buffer, and experience transitions are sequentially stored in the experience buffer and periodically overwritten for updates. However, the values are not the same for different samples; thus, Schaul et al. [43] proposed a method to provide the priority of experience transitions, and sample according to the priority of the samples. In order to rank different experience transitions according to their priority, Schaul et al. calculated the absolute value of TD error using the Q-value of the outputs of two networks, which was used to measure the degree of priority learning. The larger the TD error result, the more the sample needs to be learned, that is, the more high the priority. The sampling distribution is shown in Equation (9):

(9) $$p_{t}\propto {\left|r_{t+1}+\gamma_{t+1}max_{a_{t+1}}Q\left(s_{t+1},\;a_{t+1};\theta^{-}\right)-Q\left(s_{t},\;a_{t};\theta \right)\right|}^{\omega }$$

## 4. Problem Formulation

The LHDFJSP involves multiple smart factories located in different geographical locations, each of which may contain a varying number and types of machines. All operations of a job can be processed in the same job shop or transferred in different job shops according to their predetermined or intrinsic sequence of operations. This section provides a problem description and establishes the mathematical model of the LHDFJSP. Different from previous works that restrict all the operations of a job to the same job shop or assume homogenous job shops, this paper considers heterogeneous job shops with different machine processing capabilities and energy consumptions, different transportation times between job shops and machines, and more flexible job transfers between different job shops.

### 4.1. Problem Description

A low-carbon heterogeneous distributed flexible job shop (LHDFJ) involves multiple workshops, each of which has heterogeneous machines. Jobs arrive sequentially for processing, and each job has a set of operations that can be processed by more than one machine. Each operation of each job has a sequence of constraints.

**Figure 1:**
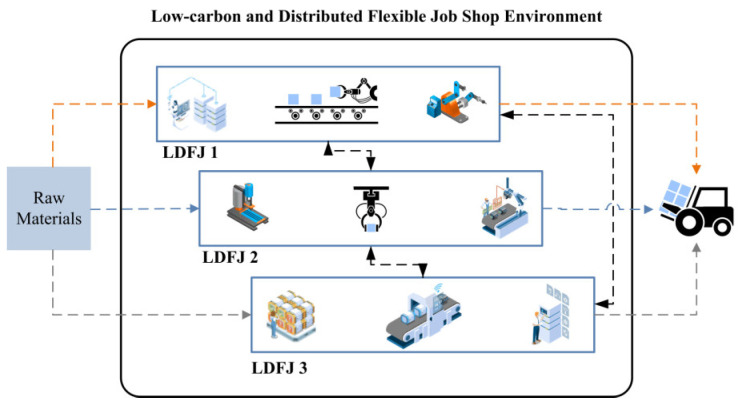
LHDFJS framework.

As shown in Figure 1, the LHDFJSP fabricates jobs through collaborative production between different LHDFJs. All operations of a job can be completed in the same LHDFJ or be transferred to different LHDFJs for processing. Different LHDFJs exhibit variations in terms of the number of machines, machine technologies, processing energy consumption, and idle energy consumption, among other factors. Efficient scheduling and resource allocation may be required to manage and coordinate the production activities of these heterogeneous workshops to ensure optimal production efficiency and product quality.

To facilitate understanding, an example of processing two jobs in two workshops is exhibited. Table 2 illustrates the processing time and energy consumption of each operation on each machine. For example, the processing time of $O_{11}$ executed by machine $m_{1}$ is 3, and the corresponding processing energy consumption is 13. The character “-” indicates that the operation cannot be processed by the machine. Table 3 exhibits the transfer time between workshops and machines. For example, the transfer time between machine $m_{1}$ in workshop 1 and machine $m_{1}$ in workshop 2 is 150, and the transfer time between machine $m_{1}$ and machine $m_{3}$ in workshop 1 is 15. The transfer of the job is divided into two cases:

1. If the preceding and succeeding operations of a job are processed in different machines of the same job shops, transport time between machines should be considered. For example, as shown in Table 3, the transport time between $m_{1}$ and $m_{2}$ in workshop 1 is 20 units of time;
2. If the preceding and succeeding operations of a job are processed in different job shops, only the transport time between different job shops is considered, while neglecting the transport time between machines. For example, as shown in Table 3, the transfer time between two workshops is fixed at 150 units of time, regardless of specific machines involved in the transition from workshop 1 to workshop 2.

The processing time, transport time, and energy consumption information are all known a priori. The objective of the LHDFJSP is to find the best processing job combination for each LHDFJ while considering the machine capacity constraints, that is, to select the optimal processing machine for each operation, and determine the optimal processing sequence of jobs on each machine in each LHDFJ, in order to minimize the total weighted tardiness and energy consumption generated during the processing. The problem is based on the following assumptions:

1. The available time of each machine is 0;
2. Loading and unloading time of jobs is not considered;
3. All operations cannot be interrupted/preempted in the processing process;
4. The machine can run continuously and there is sufficient buffer between machines;
5. There are sufficient transport devices to complete the transfer of jobs.

### 4.2. Mathematical Model

Table 4 presents the symbols used in the model (indexes all start from 1).

For the mathematical model of LHDFJS, an MILP is proposed to minimize the total weighted tardiness and total energy consumption. The MILP model consists of objective functions and constraints. LHDFJS can be formulated as follows:

1. Objective:
  (10) $$TT=\overset{N}{\underset{i=1}{\sum }}max\left(CT_{i}-D_{i},0\right)$$
  (11) TE=procE+idleE+transE

Equation (10) computes the total weighted tardiness (TT) of the LHDFJSP model based on the job information; $CT_{i}$ is computed as $CT_{i}=\overset{F}{\underset{f=1}{\sum }}\overset{M_{f}}{\underset{k=1}{\sum }}C_{i,n_{i}}\cdot x_{in_{i}}^{fk}$. Equation (11) calculates the total energy consumption during processing, including processing energy consumption, idle energy consumption of machine, and transportation energy consumption. The procE, idleE, and transE are formulated as Equations (12)–(14).

(12) $$procE=\overset{F}{\underset{f=1}{\sum }}\overset{M_{f}}{\underset{k=1}{\sum }}\overset{N}{\underset{i=1}{\sum }}\overset{n_{i}}{\underset{j=1}{\sum }}pt_{ij}^{fk}\cdot pe_{fk}\cdot x_{ij}^{fk}$$

(13) $$idleE=\overset{F}{\underset{f=1}{\sum }}\overset{M_{f}}{\underset{k=1}{\sum }}\overset{N}{\underset{i=1}{\sum }}\overset{n_{i}}{\underset{j=1}{\sum }}\overset{N}{\underset{g=1}{\sum }}\overset{n_{g}}{\underset{h=1}{\sum }}ie_{fk}\cdot \left(S_{g,h}-C_{i,j}\right)\cdot y_{ij,gh}\cdot x_{ij}^{fk}\cdot x_{gh}^{fk}$$

(14) $$transE=\overset{F}{\underset{f=1}{\sum }}\overset{F}{\underset{u=1}{\sum }}\overset{N}{\underset{i=1}{\sum }}te\cdot TransF_{fu}\cdot b_{i}^{fu}+\overset{F}{\underset{f=1}{\sum }}\overset{M_{f}}{\underset{l=1}{\sum }}\overset{M_{f}}{\underset{k=1}{\sum }}\overset{N}{\underset{i=1}{\sum }}te\cdot TransM_{lk}\cdot a_{i}^{lk}$$

2. The assumptions and constraints are as follows:
  (15) $$\overset{F}{\underset{f=1}{\sum }}\overset{M_{f}}{\underset{k=1}{\sum }}x_{ij}^{fk}=1,\forall i,j$$
  (16) $$\left(S_{i,1}-A_{i}\right)\cdot x_{i1}^{fk}\geq 0,\forall i,f,k$$
  (17) $$C_{i,j}=\left(S_{i,j}+pt_{ij}^{fk}\right)\cdot x_{ij}^{fk}$$
  (18) $$S_{i,j}\cdot x_{ij}^{fk}-TransF_{uf}\cdot b_{i}^{uf}-TransM_{lk}\cdot a_{i}^{lk}-C_{i,\left(j-1\right)}\cdot x_{i\left(j-1\right)}^{ul}\geq 0,\forall i,j,f,u,k,l,\forall j\in \left[2,N\right]$$
  (19) $$\left(S_{g,h}-C_{i,j}\right)\cdot x_{ij}^{fk}\cdot x_{gh}^{fk}\cdot y_{ij,gh}+\left(S_{i,j}-C_{g,h}\right)\cdot x_{ij}^{fk}\cdot x_{gh}^{fk}\cdot y_{gh,ij}\geq 0,\forall i,j,f,k,g,h$$
  (20) $$a_{i}^{lk}+b_{i}^{fu}\leq 1,\forall i,l,k,f,u$$
  where Constraint Equation (15) restricts that an operation can be machined in exactly one machine of one job shop. Constraint Equation (16) ensures that each job can only be processed after its arrival. Constraint Equation (17) indicates that the completion time of the operation is equal to the start time plus the processing time. Constraint Equation (18) states that the operations of each job must follow the priority order from front to back. According to Constraint Equation (19), if the operations of different jobs are to be processed by the one machine, they must be processed in sequence. Constraint Equation (20) does not take into account the transport time between machines when considering the transport time between jobs in the job shop. That is, the transportation between job shops and machines are not considered at the same time.

## 5. Rainbow DQN in LHDFJSP

In this section, a tailored Rainbow DQN applied to the LHDFJSP is explained in detail regarding four main aspects, i.e., the designs of Rainbow DQN architecture, state space, action space, and reward function, which will be introduced in Section 5.1, Section 5.2, Section 5.3, and Section 5.4, respectively.

### 5.1. Rainbow DQN Architecture

**Figure 2:**
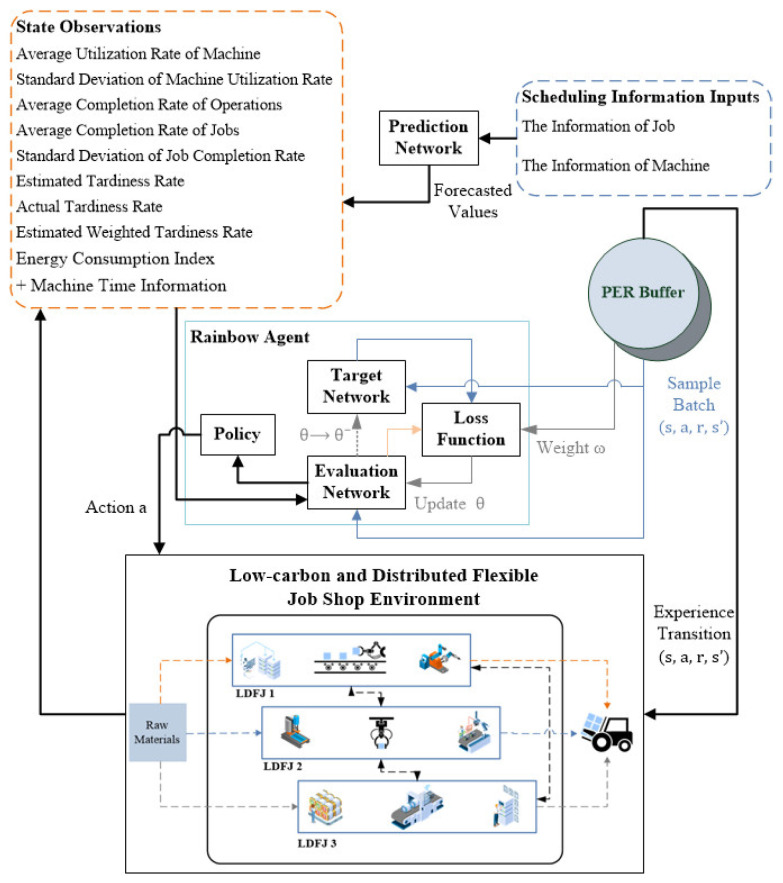
The architecture of Rainbow DQN in LDFJS.

Rainbow DQN, first proposed by Hessel et al. in 2018, incorporates various modified algorithms [44]. According to Table 1, value-based DRL methods for job shop scheduling are typically based on relatively simple DQNs or DDQNs, while Rainbow DQN represents a more powerful version. Currently, Rainbow DQN has not yet been applied in this field and whether it can contribute to solving the job shop scheduling problem is pending. Authors are curious about the application of Rainbow DQN in the field of job shop scheduling and eager to explore its performance and potential in practice. Figure 2 depicts the architecture of Rainbow DQN in LHDFJS.

Rainbow DQN takes the state of the job shop environment as input and maps it to the estimation of Q-values through a deep neural network, enabling the selection of appropriate scheduling strategies. Additionally, Rainbow DQN utilizes a prioritized experience replay mechanism to store the interaction experiences of the agent in the job shop environment. These experiences include state, chosen scheduling strategy, reward, and next state. The agent learns from past experiences to reduce data correlation and improve sample efficiency.

**Figure 3:**
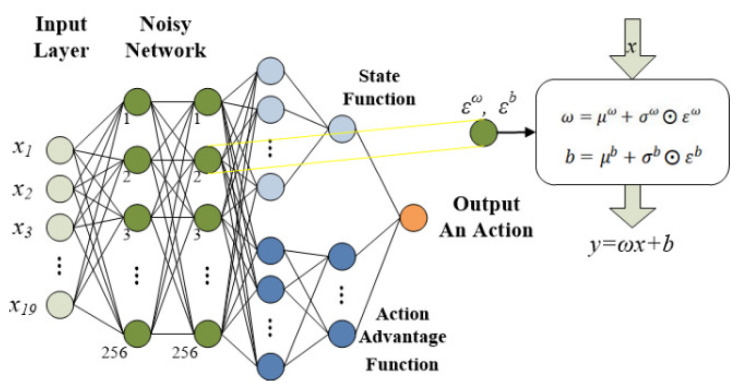
The network structure of Rainbow DQN.

In this paper, Rainbow DQN is applied to a value-based LHDFJ scheduling environment, which integrates the algorithms or concepts of double DQN, dueling DQN, noisy networks, multi-step RL, and prioritized experience replay (PER). Note that in our framework (as displayed in Figure 3), the component of distributional RL is excluded from the Rainbow DQN to accelerate the training process, since it requires more training time according to Väth and Vu [45]. After training the modified Rainbow DQN, a smart agent can make sensible decisions at each time step based on its observations of the current production state, so as to achieve satisfactory scheduling results. The overall training process is exhibited in Algorithm 1.
**Algorithm 1:** Rainbow deep Q-network1: Initialize network $Q\left(s_{t},\;a_{t};\;\theta ,\;\alpha ,\;\beta \right)$ with random weights 2: Initialize learning rate, discount factor, network parameters, replay memory 3: **For** episode n=0
**to**
N **do** 4:   Reset the state $s_{t}$ 5:   **For** t=0 **to**
T **do** 6:    Select action $a_{t}$ and execute $a_{t}$ 7:    Observe the reward $r_{t}$ and next state $s_{t+1}$ 8:    Store and sample transition ($s_{t}$, $a_{t}$, $r_{t}$, $s_{t+1}$) with $i\sim P\left(i\right)=p_{i}/\sum_{j}p_{j}$ in replay memory 9:    Calculate TD-error $\delta =\sum_{k=0}^{N-1}\gamma^{k}r_{t+k}+\gamma^{N}max_{a_{t+1}}Q\left(s_{t+N},\;a_{t+1};\;\theta^{-}\right)-Q\left(s_{t},\;a_{t};\;\theta \right)$ 10:    Update transition priority $p_{i}\leftarrow \left|\delta \right|$ 11:    Set $\theta^{-}\leftarrow \theta$ every C steps 12:   **End for** 13: **End for**

### 5.2. State Space

The state space comprehensively reflects the production status of the rescheduled points and contains a total of 10 LHDFJ state information units. To facilitate the understanding of the 10 state information units, it is necessary to clarify the parameters and formulas involved in Table 5 (indexes all start at 1).

The state space of Rainbow DQN in the context of job shop scheduling is described as follows:

1. Estimated delay rate at rescheduling point t, $Tard_{e}\left(t\right)$:
  (33) $$Tard_{e}\left(t\right)=\frac{\sum_{i\in TardJ_{e}\left(t\right)}\left(n_{i}-NPO_{i}\left(t\right)\right)}{\sum_{i\in UcompJ\left(t\right)}\left(n_{i}-NPO_{i}\left(t\right)\right)}$$

Here, $TardJ_{e}\left(t\right)$ denotes the set of jobs that are expected to be delayed at rescheduling point t and $UcompJ\left(t\right)$ denotes the set of jobs whose processing is not completed at rescheduling point t. In addition, the estimated delay of job $J_{i}$ at rescheduling point t is judged by $NPO_{i}\left(t\right)\langle n_{i}\;and\;EDT_{i}\left(t\right)\rangle 0$.

2. The actual delay rate at rescheduling point t, $Tard_{a}\left(t\right)$:
  (34) $$Tard_{a}\left(t\right)=\frac{\sum_{i\in TardJ_{a}\left(t\right)}\left(n_{i}-NPO_{i}\left(t\right)\right)}{\sum_{i\in UcompJ\left(t\right)}\left(n_{i}-NPO_{i}\left(t\right)\right)}$$

Here, $TardJ_{a}\left(t\right)$ represents the set of jobs that are actually postponed at rescheduling point t.

In addition, the actual delay of job $J_{i}$ at rescheduling point t is judged by $NPO_{i}\left(t\right)<n_{i}\;and\;C_{i,NPO_{i}\left(t\right)}\geq D_{i}$.

3. Estimated weighted delay rate at rescheduling point t, $WTard_{e}\left(t\right)$:
  (35) $$WTard_{e}\left(t\right)=\frac{\sum_{i\in TardJ_{e}\left(t\right)}\bar{T_{i}\left(t\right)}\cdot W_{i}}{\sum_{i\in UcompJ\left(t\right)}\bar{T_{i}\left(t\right)}\cdot W_{i}}$$
4. Average utilization rate of all machines in all job shops at rescheduling point t, $UR_{ave}\left(t\right)$:
  (36) $$UR_{ave}\left(t\right)=\frac{\sum_{f=1}^{F}\sum_{k=1}^{M_{f}}UR_{k}^{f}\left(t\right)}{\sum_{f=1}^{F}M_{f}}$$
5. The standard deviation of machine utilization at rescheduling point t, $UR_{std}\left(t\right)$:
  (37) $$UR_{std}\left(t\right)=\sqrt{\frac{\sum_{f=1}^{F}\sum_{k=1}^{M_{f}}{\left(UR_{k}^{f}\left(t\right)-UR_{ave}\left(t\right)\right)}^{2}}{\sum_{f=1}^{F}M_{f}}}$$
6. Average completion rate of all operations at rescheduling point t, $CRO_{ave}\left(t\right)$:
  (38) $$CRO_{ave}\left(t\right)=\frac{\sum_{i=1}^{N}NPO_{i}\left(t\right)}{\sum_{i=1}^{N}n_{i}}$$
7. Average completion rate for all jobs at rescheduling point t, $CRJ_{ave}\left(t\right)$:
  (39) $$CRJ_{ave}\left(t\right)=\frac{\sum_{i=1}^{N}CRJ_{i}\left(t\right)}{N}$$
8. The standard deviation of all job completion rate at rescheduling point t, $CRJ_{std}\left(t\right)$:
  (40) $$CRJ_{std}\left(t\right)=\sqrt{\frac{\sum_{i=1}^{N}{\left(CRJ_{i}\left(t\right)-CRJ_{ave}\left(t\right)\right)}^{2}}{N}}$$
9. The energy consumption index of all completed operations at rescheduling point t, $ECI\left(t\right)$:
  (41) $$ECI\left(t\right)=\frac{TE_{i,j}^{mid}\left(t\right)-TE_{i,j}\left(t\right)}{TE_{i,j}^{mid}\left(t\right)-TE_{i,j}^{min}\left(t\right)}$$

Here, $TE_{i,j}^{mid}\left(t\right)=\frac{TE_{i,j}^{min}\left(t\right)+TE_{i,j}^{max}\left(t\right)}{2}$.

10. The reduced completion time of the last operation processed on the machine $M_{k}^{f}$ at the rescheduling point t, $RCTM_{fk}\left(t\right)$:
  (42) $$RCTM_{fk}\left(t\right)=CTM_{k}^{f}\left(t\right)-T_{cur}$$

### 5.3. Action Space

In this paper, the classical composite scheduling rule is used as the action space. Each action consists of a job selecting rule and a machine assignment rule. Based on the state space, 7 job classification rules and 6 machine assignment rules are set, and then a total of 42 composite scheduling rules are obtained by Cartesian quadrature. Among the 42 candidate rules, the first 10 rules with good average results are chosen as the action space. Specifically, the action space of the Rainbow agent dynamically changes at each time step. According to the sizes in the dataset, the agent selects the 10 most effective rules from the 42 candidate composite scheduling rules as the action space.

#### 5.3.1. Job Selecting Rule

This subsection presents seven job selecting rules, which are described as follows:

***Job Selecting Rule 1:*** At the rescheduling point t, if $TardJ_{a}\left(t\right)$ is not an empty set, the largest ${\mathrm{EDT}}_{i}\left(t\right)\cdot W_{i}$ in ${\mathrm{TardJ}}_{a}\left(t\right)$ is chosen as the next scheduling procedure.

If $TardJ_{a}\left(t\right)$ is null, the next scheduled operation with the smallest average slack time $ST_{i}\left(t\right)$ in $UcompJ\left(t\right)$ is chosen.

(43) $$ST_{i}\left(t\right)=\frac{D_{i}-max\left(T_{cur},C_{i,NPO_{i}\left(t\right)}\right)}{n_{i}-NPO_{i}\left(t\right)}$$

***Job Selecting Rule 2:*** At the rescheduling point t, if $TardJ_{a}\left(t\right)$ is not an empty set, the largest $EDT_{i}\left(t\right)\cdot W_{i}$ in $TardJ_{a}\left(t\right)$ is chosen as the next scheduling procedure.

If $TardJ_{a}\left(t\right)$ is null, the next scheduled operation with the smallest critical ratio $CR\left(i\right)$ in $UcompJ\left(t\right)$ is chosen.

(44) $$CR\left(i\right)=\frac{D_{i}-max\left(T_{cur},C_{i,NPO_{i}\left(t\right)}\right)}{\bar{T_{i}\left(t\right)}}$$

***Job Selecting Rule 3:*** Based on $T_{cur}$, the jobs are sorted according to the estimated weighted tardiness $EDT_{i}\left(t\right)\cdot W_{i}$, and the one with the largest $EDT_{i}\left(t\right)\cdot W_{i}$ is selected as the next scheduling procedure. If there are multiple identical values, choose one randomly.

***Job Selecting Rule 4:*** Select a random job from $UcompJ\left(t\right)$.

***Job Selecting Rule 5:*** At rescheduling point t, if $TardJ_{a}\left(t\right)$ is not an empty set, the maximum $\frac{n_{i}-NPO_{i}\left(t\right)}{\sum_{i=1}^{N}\left(n_{i}-NPO_{i}\left(t\right)\right)}\cdot \bar{T_{i}\left(t\right)}\cdot W_{i}$ (critical ratio of weighted tardiness) in $TardJ_{a}\left(t\right)$ is chosen as the next scheduling procedure.

If $TardJ_{a}\left(t\right)$ is null, the next scheduled operation is that with the smallest $\frac{NPO_{i}\left(t\right)}{n_{i}}\cdot \left(D_{i}-max\left(T_{cur},C_{i,NPO_{i}\left(t\right)}\right)\right)$ in $UcompJ\left(t\right)$ is chosen.

***Job Selecting Rule 6:*** At rescheduling point t, select the job with the lowest completion rate in $UcompJ\left(t\right)$.

***Job Selecting Rule 7:*** At rescheduling point t, assign priority based on the deadline of the jobs. The earlier the deadline, the higher the processing priority.

#### 5.3.2. Machine Assignment Rule

This subsection presents six machine assignment rules, which are described as follows:

***Machine Assignment Rule 1:*** Select the earliest available machine $m_{k}$.

(45) $$k=argmin_{k\in M_{ij}^{f},f\in F_{i,j}}\left(max\left(CT_{k}\left(t\right),C_{i,j-1}+T_{k}^{f}\right)\right)$$

Here, $T_{k}^{f}$ is the transportation time from the previous operation to machine $m_{k}$ in job shop f.

***Machine Assignment Rule 2:*** Select the available machine $m_{k}^{f}$ with the lowest energy consumption (transportation energy consumption plus processing energy consumption plus idle energy consumption), $k=TE_{i,j}^{min}\left(t\right)$ (see state space parameters for the calculation method).

***Machine Assignment Rule 3:*** Select the available machine $m_{k}^{f}$ with the lowest utilization rate of machine $UR_{k}^{f}\left(t\right)$.

(46) $$UR_{k}^{f}\left(t\right)=\frac{\sum_{i=1}^{n}\sum_{j=1}^{NPO_{i}\left(t\right)}pt_{ij}^{fk}\cdot x_{ij}^{fk}}{CT_{k}^{f}\left(t\right)}$$

***Machine Assignment Rule 4:*** Select the available machine with the shortest processing time.

***Machine Assignment Rule 5:*** Select the machine that is available and has the shortest processing time for the previous operation.

***Machine Assignment Rule 6:*** Choose the available machine with the minimum number of expected usage in all operations of the next round.

### 5.4. Reward Function

In the context of the LHDFJSP, conflicting optimization objectives arise. For instance, the scheduling scheme aims to minimize both the total weighted tardiness and the total energy consumption. While minimizing the total weighted tardiness implies reducing the processing time on machines, which results in higher energy consumption, minimizing the total energy consumption requires lower energy consumption during product processing. As a result, the agent’s policy cannot optimize all objectives but rather learns a policy that achieves a better outcome among the conflicting ones.

In this paper, the reward function includes immediate reward function $R_{t}$ and episodic reward function ER; the formula is presented below.

#### 5.4.1. Immediate Reward Function

The immediate reward function consists of three components: the economic index, the energy consumption index, and the machine index.

The calculation formula of economic index reward $eco_{t}$:

(47) $$eco_{t}=eco_{tarda}+eco_{wtard}+eco_{tarde}+eco_{ur}+eco_{tardc}$$

Here, $eco_{tarda}$ represents the reward given based on the actual tardiness rate $Tard_{a}\left(t\right)$ of current state and next state. $eco_{wtard}$ represents the reward given based on the estimated weighted tardiness rate $WTard_{e}\left(t\right)$ of the current state and next state. $eco_{tarde}$ represents the reward given based on the estimated tardiness rate $Tard_{e}\left(t\right)$ of current state and next state. $eco_{ur}$ represents the reward given based on the average utilization rate of machine $UR_{ave}\left(t\right)$ of the current state and next state. $eco_{tardc}$ represents the reward calculated based on the minimum total weighted tardiness and the current total weighted tardiness during training. The calculation formula is as follows:

(48) $$eco_{tarda}=\left\{\begin{array}{c} 1,\;Tard_{a}\left(t+1\right)<Tard_{a}\left(t\right)\\ -1,\;Tard_{a}\left(t+1\right)>Tard_{a}\left(t\right)\\ 0,\;Tard_{a}\left(t+1\right)=Tard_{a}\left(t\right) \end{array}\right.$$

(49) $$eco_{wtard}=\left\{\begin{array}{c} 1,\;WTard_{e}\left(t+1\right)<WTard_{e}\left(t\right)\\ -1,\;WTard_{e}\left(t+1\right)>WTard_{e}\left(t\right)\\ 0,\;WTard_{e}\left(t+1\right)=WTard_{e}\left(t\right) \end{array}\right.$$

(50) $$eco_{tarde}=\left\{\begin{array}{c} 1,\;Tard_{e}\left(t+1\right)<Tard_{e}\left(t\right)\\ -1,\;Tard_{e}\left(t+1\right)>Tard_{e}\left(t\right)\\ 0,\;Tard_{e}\left(t+1\right)=Tard_{e}\left(t\right) \end{array}\right.$$

(51) $$eco_{ur}=\left\{\begin{array}{c} 1,\;UR_{ave}\left(t+1\right)<UR_{ave}\left(t\right)\\ -1,\;UR_{ave}\left(t+1\right)>UR_{ave}\left(t\right)\\ 0,\;UR_{ave}\left(t+1\right)=UR_{ave}\left(t\right) \end{array}\right.$$

(52) $$eco_{tardc}=\left\{\begin{array}{c} 0,\;minTard=currentTard\\ 1,\;minTard<currentTard\\ -50,\;minTard>=currentTard \end{array}\right.$$

where t represents the current rescheduling point or decision point and t+1 represents the next rescheduling point or decision point.

Calculation formula of energy consumption index reward $ene_{t}$:

(53) $$ene_{t}=ene_{ECI}+ene_{CE}$$

Here, $ene_{ECI}$ represents the reward given based on the energy consumption index $ECI\left(t\right)$ of the current state and next state. $ene_{CE}$ represents the reward calculated based on the minimum total energy consumption and the current total energy consumption during training. The calculation formula is as follows:

(54) $$ene_{ECI}=\left\{\begin{array}{c} 1,\;ECI\left(t\right)>ECI\left(t+1\right)\\ -1,\;ECI\left(t\right)<ECI\left(t+1\right)\\ 0,\;ECI\left(t\right)=ECI\left(t+1\right) \end{array}\right.$$

(55) $$ene_{CE}=\left\{\begin{array}{c} 0,\;minEnergy=currentEnergy\\ 1,\;minEnergy<currentEnergy\\ -50,\;minEnergy>=currentEnergy \end{array}\right.$$

The weighted sum of the economic index and the energy consumption index is used as the immediate reward of the rescheduled point; parameter $\beta \in \left[0,1\right]$ is used to balance economic index and energy consumption index:

(56) $$R_{t}=\beta \cdot eco_{t}+\left(1-\beta \right)\cdot ene_{t}$$

The reward function of machine index refers to the negative reward given to the reduced completion time $RCTM_{fk}\left(t\right)$ of the last operation on all machines and is fed back to the agent with a strongly correlated negative reward value; then, it facilitates the agent achieving faster convergence and better convergence effect.

(57) $$R_{t}=\sum_{f=1}^{F}\sum_{k=1}^{M_{f}}RCTM_{fk}\left(t\right)$$

#### 5.4.2. Episodic Reward Function

The episodic reward is a negative value. The LHDFJ computes the total weighted tardiness $CT_{episode}$ and the total energy consumption $TE_{episode}$ after each episode. The larger these two values are the greater penalty the environment will feed back to the agent. The parameters ρ and φ are used to reduce the overall value of the objective and match the previous reward values.

(58) $$ER=-\left(CT_{episode}\cdot \rho +TE_{episode}\cdot \varphi \right)$$

## 6. Experiments

In this section, comprehensive experiments are conducted to evaluate the Rainbow DQN framework with composite scheduling rules. Section 6.1 and Section 6.3 introduce the generation of problem instances and the algorithms for comparison. Section 6.2 primarily elucidates the details of algorithm training. Section 6.4 exhibits comparative results of various scheduling strategies, through which the effectiveness and performance advantages of the proposed Rainbow DQN-based method could be validated. All the experiments were carried out on an AMD Ryzen 5 5600X 6-Core Processor @ 3.70 GHz with 32 G RAM and an NVIDIA GeForce RTX 3060. Additionally, experiments were conducted using Python 3.8, with the main libraries including PyTorch 1.11.0 and NumPy 1.20.0.

### 6.1. Generation of Problem Instances

To date, there is no public dataset that takes into account complicated factors including job insertions, energy consumption, and transportation of heterogeneous distributed job shops. Therefore, this study extended the existing benchmarks and utilized these problem instances to validate the applicability of the proposed method and evaluate its performance under different scenarios.

The problem instances are extended based on the *mk* series datasets proposed by Brandimarte [46]. A total of 8 experimental scenarios were designed for evaluation. Detailed parameter settings are listed in Table 6, including the numbers of job shops, initial jobs, dynamically inserted jobs, and machines. Furthermore, Table 7 indicates the general parameters for different scenarios, i.e., the number of operations for each problem instance, transportation time between machines or job shops, unit processing energy consumption, unit idle energy consumption, unit transport energy consumption, DDT, and the weights of jobs.

The arrival interval Y between two consecutive dynamically inserted jobs obeys the exponential distribution $Y\sim exp\left(1/\lambda \right)$; the λ is set to 50. In addition, the due date of job $J_{i}$, denoted by $D_{i}$, can be calculated according to DDT, and the calculation formula is as follows:

(59) $$D_{i}=A_{i}+\left(\sum_{j=1}^{n_{i}}\bar{pt_{ij}^{fk}}\right)\cdot DDT$$

Here, $\bar{pt_{ij}^{fk}}$ represents the average processing time of operation $O_{ij}$ on all available machines in all job shops.

(60) $$\bar{pt_{ij}^{fk}}=mean\left(\sum_{f=1}^{F}\sum_{k\in 1}^{M_{f}}pt_{ij}^{fk}\right)$$

### 6.2. Training Details

During the training process, adjustments were made to several Rainbow DQN parameters to enhance convergence speed and effectiveness in job shop scheduling. The recommended hyperparameters for the Rainbow DQN are listed in Table 8. The selection of these hyperparameters has been carefully scrutinized to better adapt to the complexity of scheduling during the training process. The hyperparameters in this paper were manually adjusted based on experience and domain knowledge. Initially, the generic Rainbow DQN hyperparameters were used, and then manually fine-tuned according to modifications in the scheduling environment. This process involved iterative adjustments and testing of each hyperparameter to observe its impact on the algorithm’s performance, ultimately selecting the optimal parameter combination.

**Figure 4:**
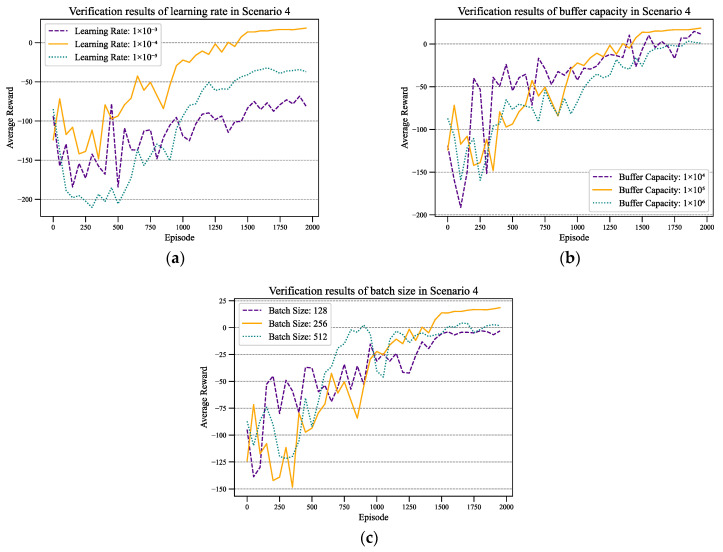
Verification results of hyperparameters: (**a**) learning rate; (**b**) buffer capacity; (**c**) batch size.

Figure 4 illustrates the convergence performance of the Rainbow DQN algorithm under different hyperparameter settings. Specifically, we focus on the impact of different learning rates, batch sizes, and buffer capacities on the algorithm across various metrics. This allows for us to determine the algorithm’s convergence speed, efficiency, and ultimate stability.

As shown in Figure 4a, under different learning rates, the trend of the average reward curves remains consistent, but the orange curve (with the learning rate equal to $1\times 10^{-4}$) consistently achieves higher reward values throughout most episodes compared to the other curves. Thus, the learning rate is set to $1\times 10^{-4}$.

Figure 4b illustrates the impact of buffer size on average rewards. When the buffer size is 1 × 10^5^, the convergence speed is relatively slow in the first half, but the convergence efficiency is higher in the latter half, resulting in a more stable convergence outcome.

Figure 4c summarizes the convergence scenarios for different batch sizes, indicating that a batch size of 256 leads to superior convergence effectiveness and efficiency.

The experimentation and adjustment of hyperparameters contribute to a deeper understanding of the performance of Rainbow DQN in job shop scheduling. Furthermore, it provides guidance for further optimization and tuning. Through a systematic validation of different parameter combinations, a better understanding of the algorithm’s robustness can be gained, thereby better meeting the requirements of real-world production environments.

### 6.3. Algorithms for Comparison

According to Lei et al. [47], the top four job assignment scheduling rules and two machine assignment scheduling rules were selected, and eight heuristic scheduling rules were obtained by Cartesian quadrature (shown in Table 9). These rules are considered as the benchmarks for evaluation.

This paper introduces 42 candidate composite scheduling rules specifically designed for the LHDFJSP and employs the Rainbow DQN framework to select the optimal scheduling rule at each decision point. In order to evaluate the performance of the presented method, a comparison is conducted between Rainbow DQN and the 8 classical scheduling rules, as well as the 42 composite scheduling rules, using different problem instances.

Furthermore, to demonstrate the superior learning capability of Rainbow DQN, we also compare the Rainbow DQN-based method and dueling double DQN with prioritized experience replay (D3QN with PER), which combines the advantages of double DQN and dueling DQN with prioritized experience replay by improving the network architecture and experience sampling to reduce overestimation bias, enhance exploration capability, and improve learning efficiency. These experiments allow for a comparison of the proposed Rainbow DQN method with established scheduling rules in the LHDFJSP domain.

### 6.4. Experimental Results

This subsection evaluates the performance of the Rainbow DQN with composite scheduling rules. The weight β in Formula (56) is set to 0.7, signifying a prioritization of 0.7 of the economic goal and 0.3 for the environmental goal. This choice underscores a greater emphasis on time efficiency while still maintaining the importance of energy consumption. The parameters ρ and φ in the episodic reward function (58) are primarily used for value scaling and are set to 160 and 1600, respectively, to ensure the magnitude of the reward values aligns with the values of other reward functions.

#### 6.4.1. Comparison of Different Algorithms

Table 10 exhibits performance comparison of classical scheduling rules and composite scheduling rules in various scenarios. Each row indicates a scenario, while each column stands for a scheduling algorithm. In the table, only four sets of classical scheduling rules are exhibited, as they achieved top performance among all the eight rules. The column of the composite scheduling rules shows the optimal value selected from a pool of 42 candidate composite scheduling rules. A bold value represents the best solution, and an underlined value indicates the second best solution. The last column, i.e., solution distance, represents the deviation between the best solution obtained by composite scheduling rules and the currently acquired best solution (bold value). From the solution distance, it can be observed that the composite scheduling rules consistently achieve the optimal solution for the total weighted delay metric in all the scenarios. However, in terms of the total energy consumption metric, there is still a gap between the composite scheduling rules and other classical heuristic scheduling rules, except for Scenario 1.

Table 11 exhibits the comparison results of classical scheduling rules, composite scheduling rules, D3QN with PER, and Rainbow DQN. In Scenarios 2, 3, 4, and 6, Rainbow DQN exhibits significantly lower total weighted tardiness and total energy consumption values compared to other algorithms. Even in more challenging scenarios such as Scenario 7 and Scenario 8, the Rainbow DQN algorithm maintains a competitive edge by achieving relatively lower total weighted tardiness values. Rainbow DQN achieves a solution distance of 0 in most scenarios, indicating that its solutions closely match the optimal solutions, highlighting its ability to approach optimality.

Although Rainbow DQN may exhibit slightly higher total energy consumption values in certain scenarios (such as Scenarios 5 and 8), it is important to consider the tradeoff between energy consumption and job tardiness. In some cases, Rainbow DQN might prioritize minimizing tardiness over energy consumption, which could lead to marginally higher energy usage. However, this tradeoff is reasonable as it ensures timely job completion and prevents potential penalties associated with job delays.

Overall, the Rainbow DQN algorithm demonstrates its superiority in terms of total weighted tardiness across all scenarios, indicating its ability to optimize job scheduling effectively in LHDFJSP. While total energy consumption may be slightly higher in some cases, Rainbow DQN still provides competitive results and offers a promising solution for addressing the challenges of the LHDFJSP in terms of both job tardiness and energy consumption. This demonstrates its robustness and adaptability in handling complex scheduling problems. Rainbow DQN proves to be a reliable solution for the LHDFJSP, capable of optimizing both production timelines and energy consumption.

#### 6.4.2. Optimization Process of Rainbow DQN

**Figure 5:**
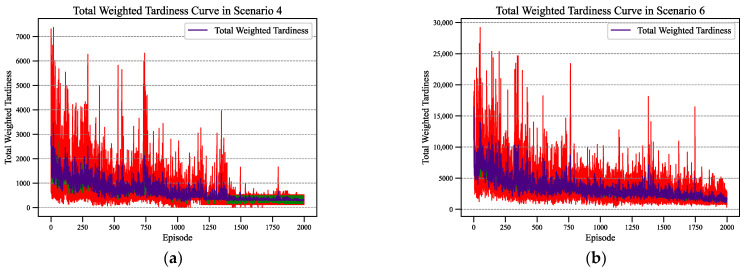
The total weighted tardiness curve of Rainbow DQN in two scenarios: (**a**) Scenario 4; (**b**) Scenario 6.

To demonstrate the adaptability of Rainbow DQN in different sizes of problem instances, we depicted the optimization process curves of Rainbow DQN when optimizing the total weighted tardiness and total energy consumption in Scenarios 4 and 6, respectively, with varying numbers of jobs. Figure 5 depicts the variations in total weighted tardiness during the training process of Rainbow DQN during 2000 rounds of iterative training. The red area represents the upper and lower bounds on the weighted tardiness of Rainbow DQN over multiple training sessions, while the indigo curve represents the average weighted tardiness per episode. From the changing curves, it can be observed that in the initial stages of training, there is a higher total weighted tardiness. This is because the agent randomly selects actions during the early exploration phase to collect transition experiences by interacting with the scheduling environment. As the priority experience replay buffer reaches its maximum capacity, the agent gradually learns to optimize its strategy, resulting in a gradual reduction and convergence of total weighted tardiness.

**Figure 6:**
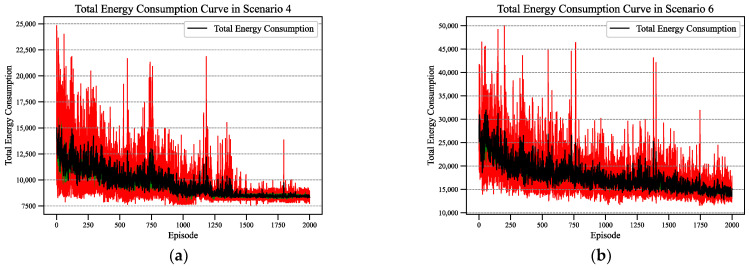
The energy consumption curve of Rainbow DQN in two scenarios: (**a**) Scenario 4; (**b**) Scenario 6.

Similarly, Figure 6 represents the variations in total energy consumption during the training process of the Rainbow DQN algorithm in Scenarios 4 and 6. From the change curves, it can be observed that in the initial stages of training, there is higher total energy consumption. Afterwards, as the agent gradually learns to optimize its strategy, the total energy consumption decreases and converges over time.

#### 6.4.3. Optimization Process of different DRL Methods

**Figure 7:**
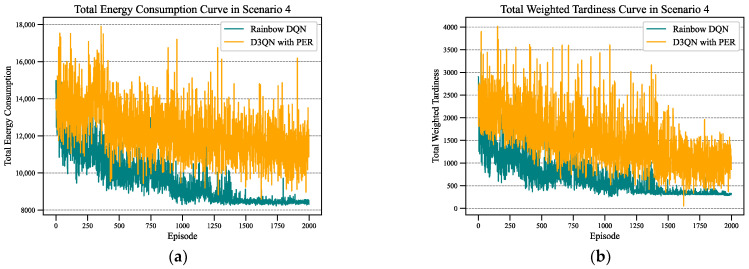
Rainbow DQN versus D3QN with PER in Scenario 4: (**a**) total energy consumption curve; (**b**) total weighted tardiness curve.

In this subsection, Scenario 4 is taken as an example to compare the training patterns of Rainbow DQN and D3QN when applied to the LHDFJSP. As displayed in Figure 7, while both algorithms exhibit a downward trend in their training curves, there are notable differences in their characteristics. The D3QN with PER training curve shows a gradual decrease over time, indicating a gradual improvement in performance. However, the curve also exhibits significant fluctuations, suggesting a certain degree of instability; the training curve of Rainbow DQN displayed a comparatively smoother trajectory. The curve exhibited a faster convergence and demonstrated a more consistent decrease in the objective metric, indicating improved performance over time. This instability during the former’s training process compared to the smoothness in the latter’s training curve suggests that Rainbow DQN was able to learn more stable and reliable strategies during training. Overall, comparing D3QN with PER and Rainbow DQN, it becomes evident that the latter exhibits greater robustness and reliability, leading to superior performance. The differences in their training curves highlight the advantages of Rainbow DQN in terms of stability and consistency.

In summary, the comparison between classic heuristic scheduling rules and Rainbow DQN reveals that the latter consistently outperforms the former in terms of both total weighted tardiness and total energy consumption across different scenarios. Classic heuristic scheduling rules, although widely used and established, often rely on heuristics or predefined strategies that may not adapt well to dynamic and complex scheduling problems like the LHDFJSP. In contrast, Rainbow DQN leverages DRL techniques to learn optimal scheduling policies from experience, allowing for it to adapt and optimize performance based on the specific problem at hand. Additionally, the composited scheduling rules, which combine multiple classic scheduling rules, provide some improvement over the heuristic rules. However, Rainbow DQN surpasses even the composited rules, achieving lower values. This demonstrates the ability of Rainbow DQN to effectively learn and optimize scheduling decisions, surpassing the performance achieved by manual combinations of classic rules. Furthermore, Rainbow DQN exhibits a more stable and reliable training curve, showcasing a smoother convergence towards optimal performance. In contrast, D3QN with PER exhibits higher volatility and fluctuations in its training curve, indicating potential challenges in achieving consistent and robust performance.

Therefore, Rainbow DQN is a superior algorithm in terms of performance. Its adaptability, robustness, and reliable performance in the LHDFJSP make it an effective approach for solving complex scheduling problems in real-world applications.

## 7. Conclusions

The characteristics of advanced technology, green concept, and new business model have brought serious challenges to the optimization and control in the field of job shop scheduling. In this paper, the mathematical model of the low-carbon heterogeneous distributed flexible job shop scheduling (LHDFJS) problem and a solution based on DRL were proposed to fill in some shortcomings in the field of scheduling.

Aiming at the DMoSP for LHDFJS, a multi-objective mathematical model was established with the objective of minimizing the total weighted tardiness and total energy consumption of the processing process. In this context, a set of composite scheduling rules were designed by combining job selecting rules and machine assignment rules. To select the optimal scheduling rule at each decision point, a Rainbow DQN framework was employed, which redesigns the state, action, and reward components to capture the production status and balance economic and environmental considerations. Comparative experiments were carried out on a customized dataset, demonstrating that, relative to composite scheduling rules, the Rainbow DQN achieved excellent performance in minimizing both total weighted tardiness and total energy consumption.

From an academic perspective, this paper helps fill research gaps in the field and promotes the development of job shop scheduling theory. By introducing real production factors such as low-carbon targets and heterogeneity, this study enriches the research content of scheduling problems, enhancing their practicality and applicability. Importantly, from a managerial standpoint, addressing this issue can optimize production processes, improve efficiency, and reduce energy consumption, thereby lowering production costs and enhancing the competitiveness of enterprises. Additionally, the various composite scheduling rules and DRL algorithms proposed during the research process provide enterprises with new management tools and methods.

Although this study has achieved certain results, there are still limitations. For example, in reinforcement learning, the manual implementation of state features, scheduling rules, and reward functions may be limiting when dealing with massive quantities of job shop data, constraining the effectiveness of the solutions. Therefore, exploring self-learning methods based on big data to automatically generate scheduling rules holds promise for further enhancing the quality and efficiency of solutions.

For future research, the main considerations involve scheduling problems and scheduling methods. Regarding scheduling problems, there still exists a certain gap between existing studies and practical application scenarios. For example, factors such as assembly processes, labor resources, and material supply can be considered. From the perspective of scheduling methods, DRL methods in the field of job shop scheduling are still in the early exploration stage. It is possible to explore the construction of multi-agent interactions with the environment and also to attempt integrating graph neural networks or knowledge graphs to obtain higher quality solutions.

## Figures and Tables

**Table 1:** Summary of relevant studies.

References	Type of Problem	Low-Carbon	Heterogeneity	Dynamics	Transfer	Objective	Method
[11]	Flexible job shop	**✗** ^1^	**✗**	**✓**	**✗**	S ^2^	DQN
[32]	Flexible job shop	**✗**	**✗**	**✓**	**✗**	M	Improved PPO
[33]	Flexible job shop	**✗**	**✗**	**✓**	**✗**	M	Hierarchical DQN
[10]	Distributed assembly No-idle flow-shop	**✗**	**✗**	**✗**	**✗**	S	Q-learning and metaheuristic algorithm
[8]	Distributed flexible job shop	**✓**	**✗**	**✗**	**✗**	M	Metaheuristic algorithm
[15]	Distributed flexible job shop	**✗**	**✗**	**✗**	**✗**	S	Metaheuristic algorithm
[13]	Distributed flexible job shop	**✓**	**✓**	**✗**	**✓**	M	Metaheuristic algorithm
[9]	Flexible job shop	**✗**	**✗**	**✓**	**✗**	S	Monte Carlo tree search
[26]	Flexible job shop	**✗**	**✗**	**✓**	**✗**	S	Improved Q-learning
[27]	Flexible job shop	**✗**	**✗**	**✓**	**✗**	S	Double DQN
[34]	Flexible job shop	**✗**	**✗**	**✓**	**✗**	S	Double DQN
[28]	Distributed flexible permutation flow shop	**✗**	**✗**	**✗**	**✗**	S	Improved DQN
[29]	Job shop	**✗**	**✗**	**✓**	**✗**	S	Proximal policy optimization
[25]	Flexible job shop	**✓**	**✗**	**✓**	**✗**	M	Hybrid DQN
[30]	Semiconductor fabrication	**✗**	**✗**	**✓**	**✗**	M	Improved DQN
[21]	Distributed flexible job shop	**✓**	**✗**	**✓**	**✓**	M	Metaheuristic algorithm
[23]	Distributed flexible job shop	**✓**	**✗**	**✗**	**✓**	M	Metaheuristic algorithm
[14]	Distributed flexible Job shop	**✓**	**✗**	**✗**	**✓**	M	Q-learning and metaheuristic algorithm
Ours	Distributed heterogeneous flexible job Shop	**✓**	**✓**	**✓**	**✓**	M	Rainbow DQN

^1^ **✓**/**✗** denotes whether the literature includes this research direction. ^2^ S/M indicates whether the problem discussed is single-objective (S) or multi-objective (M).

**Table 2:** Exemplified processing time and energy consumption.

		Workshop 1			Workshop 2		
		$m_{1}$	$m_{2}$	$m_{3}$	$m_{1}$	$m_{3}$	$m_{4}$
$J_{1}$	$O_{11}$	3.13	-	5.9	3.13	5.9	-
	$O_{12}$	-	2.14	1.17	-	1.17	3.9
$J_{2}$	$O_{21}$	10.7	9.10	12.9	10.7	12.9	-
	$O_{22}$	7.14	11.8	-	7.14	-	6.10
	$O_{23}$	6.11	-	4.12	6.11	4.12	7.9

**Table 3:** Exemplified transfer time between workshops and machines.

		Workshop 1			Workshop 2		
		$m_{1}$	$m_{2}$	$m_{3}$	$m_{1}$	$m_{3}$	$m_{4}$
**Workshop 1**	$m_{1}$	0	20	15	150	150	150
	$m_{2}$	20	0	25	150	150	150
	$m_{3}$	15	25	0	150	150	150
**Workshop 2**	$m_{1}$	150	150	150	0	33	27
	$m_{3}$	150	150	150	33	0	19
	$m_{4}$	150	150	150	27	19	0

**Table 4:** The notation of mathematical model.

Parameter	Description	Parameter	Description
i, g	Index of jobs	$S_{i,j}$	The start time of operation $O_{ij}$
j, h	Index of operations	$C_{i,j}$	The completion time of operation $O_{ij}$
f, u	Index of job shops	$TransF_{fu}$	The transport time of job from job shop f to job shop u
k, l	Index of machines	$TransM_{lk}$	The transport time of job from machine l to machine k
N	The total number of jobs	procE	The processing energy consumption of all machines
$n_{i}$	The total number of operations for job $J_{i}$	idleE	The idle energy consumption of all machines
$O_{ij}$	The jth operation of job $J_{i}$	transE	The transport energy consumption of all transportation missions
F	The total number of job shop	TE	Total energy consumption
$M_{f}$	Total number of machines in job shop f	$pe_{fk}$	Unit processing energy consumption of machine k in job shop f
$F_{i,j}$	The set of job shops that can process operation $O_{ij}$	$ie_{fk}$	Unit idle energy consumption of machine k in job shop f
$M_{ij}^{f}$	The set of machines in job shop f that can process operation $O_{ij}$	te	Unit transport energy consumption between job shops/machines
$W_{i}$	The weight coefficient of job $J_{i}$	$x_{ij}^{fk}$	0–1 decision variable: if $O_{ij}$ is processed on machine k in job shop f, the value is 1; otherwise, it is 0.
$pt_{ij}^{fk}$	The processing time of operation $O_{ij}$ on machine k in job shop f	$y_{ij,gh}$	0–1 decision variable: if the rear operation of $O_{ij}$ is $O_{gh}$, the value is 1; otherwise, it is 0.
$A_{i}$	The arrival time of job $J_{i}$	$a_{i}^{lk}$	0–1 decision variable: if job $J_{i}$ is transported from machine l to machine k in same job shop, the value is 1; otherwise, it is 0.
$D_{i}$	The delivery date of job $J_{i}$	$b_{i}^{fu}$	0–1 decision variable: if job $J_{i}$ is transported from job shop f to job shop u, the value is 1; otherwise, it is 0.

**Table 5:** The parameters and formulas of state space.

Parameter	Description and Formulas		Value Range
t	Rescheduling point (decision point): the scheduling environment changes to a new state after scheduling each operation, namely, rescheduling point t in the DRL agent.		[0, $\sum_{i=0}^{N}n_{i}$]
$NPO_{i}\left(t\right)$	At rescheduling point t, the number of completed operations for job $J_{i}$.		[0, $n_{i}$]
$CTM_{k}^{f}\left(t\right)$	At rescheduling point t, the completion time of the last operation processed on the machine $M_{k}$.		[0, $CT_{i}$]
$T_{cur}$	At rescheduling point t, the mean completion time of the last operation assigned to each machine in each job shop. The formula is shown below:		[0, $CT_{i}$]
	$T_{cur}=\frac{\sum_{f=1}^{F}\sum_{k=1}^{M_{f}}CTM_{k}^{f}\left(t\right)}{\sum_{f=1}^{F}M_{f}}$	(21)	
$CT_{k}^{f}\left(t\right)$	At rescheduling point t, the completion time of the last operation on the machine $M_{k}$ in job shop f.		[0, $CT_{i}$]
$\bar{pt_{ij}}$	The average processing time of operation $O_{ij}$ on all available machines in all job shops.		[0, $\bar{\sum_{f=1}^{F}\sum_{k\in 1}^{M_{f}}pt_{ij}^{fk}}$]
$\bar{tt_{i,j}}(j>1)$	The average transport time from operation $O_{i,\;j-1}$ to $O_{ij}$. $\max tt_{i,j}$ represents the maximum transit time in the operation $O_{ij}$. The formula is shown below:		[0, $\max tt_{i,j}$]
	$\bar{tt_{i,j}}=\frac{1}{\left|F_{i,j}\right|}\sum_{u\in F_{i,j}}TransF_{fu}$	(22)	
$\bar{TT_{i}\left(t\right)}$	At rescheduling point t, remaining estimated transport time for job $J_{i}$. The formula is shown below:		[0, *(*$n_{i}-1)\cdot TransF_{fu}\cdot te$]
	$\bar{TT_{i}\left(t\right)}=\bar{tt_{i,NPO_{i}\left(t\right)+1}}+\sum_{j=NPO_{i}\left(t\right)+2}^{n_{i}}\frac{1}{\left|F_{i,j}\right|\cdot \left|F_{i,j+1}\right|}\sum_{f\in F_{i,j}}\sum_{u\in F_{i,j+1}}TransF_{fu}$	(23)	
$\bar{T_{i}\left(t\right)}$	At rescheduling point t, the estimated time required to process the remaining part of job $J_{i}$ (remaining process time of job $J_{i}$ + remaining transport time of job $J_{i}$). The formula is shown below:		- ^1^
	$\bar{T_{i}\left(t\right)}=\sum_{j=NPO_{i}\left(t\right)+1}^{n_{i}}\bar{pt_{ij}}+\bar{TT_{i}\left(t\right)}$	(24)	
$EDT_{i}\left(t\right)$	At rescheduling point t, the expected delayed time (EDT) of job $J_{i}$. The larger the EDT value is, the more serious the delay will be. The formula is shown below:		-
	$EDT_{i}\left(t\right)=max\left(T_{cur},C_{i,NPO_{i}\left(t\right)}\right)+\bar{T_{i}\left(t\right)}-D_{i}$	(25)	
$CRJ_{i}\left(t\right)$	At rescheduling point t, the completion rate of job $J_{i}$. The formula is shown below:		[0, 1]
	$CRJ_{i}\left(t\right)=\frac{NPO_{i}\left(t\right)}{n_{i}}$	(26)	
$UR_{k}^{f}\left(t\right)$	At rescheduling point t, utilization rate of machine $M_{k}^{f}$ in job shop f. The formula is shown below:		[0, 1]
	$UR_{k}^{f}\left(t\right)=\frac{\sum_{i=1}^{n}\sum_{j=1}^{NPO_{i}\left(t\right)}pt_{ij}^{fk}\cdot x_{ij}^{fk}}{CT_{k}^{f}\left(t\right)}$	(27)	
$TE_{i,j}\left(t\right)$	At rescheduling point t, the actual energy consumption required to complete operation $O_{ij}$.		[$TE_{i,j}^{min}\left(t\right)$, $TE_{i,j}^{max}\left(t\right)$]
$TE_{i,j}^{min}\left(t\right)$	At rescheduling point t, the minimum energy consumption required to complete operation $O_{ij}$. The formula is shown below:		-
	$TE_{i,j}^{min}\left(t\right)=min_{u\in F_{i,j},l\in M_{i,j}^{u}}\left(transE_{i,j}\left(t\right)+procE_{i,j}\left(t\right)+idleE_{i,j}\left(t\right)\right)$	(28)	
	$transE_{i,j}\left(t\right)=\left(TransF_{fu}\cdot b_{i}^{fu}+TransM_{kl}\cdot a_{i}^{kl}\right)\cdot te$	(29)	
	$procE_{i,j}\left(t\right)=pt_{ij}^{ul}\cdot pe_{ul}$	(30)	
	$idleE_{i,j}\left(t\right)=\left(max\left(C_{i,j-1}+TransF_{fu}\cdot b_{i}^{fu}+TransM_{kl}\cdot a_{i}^{kl},CT_{l}^{u}\left(t\right)\right)-CT_{l}^{u}\left(t\right)\right)\cdot ie_{ul}$	(31)	
$TE_{i,j}^{max}\left(t\right)$	At rescheduling point t, the maximum energy consumption required to complete operation $O_{ij}$. The formula is shown below:		-
	$TE_{i,j}^{max}\left(t\right)=max_{u\in F_{i,j},l\in M_{i,j}^{u}}\left(transE_{i,j}\left(t\right)+procE_{i,j}\left(t\right)+idleE_{i,j}\left(t\right)\right)$	(32)	

^1^ “-” indicates that this parameter needs to be calculated based on the overall scheduling situation.

**Table 6:** The size of problem instance.

Scenario	Number of Job Shops	Number of Initial Jobs	Number of Dynamically Inserted Jobs	Number of Machines
**1**	3	10	5	10
**2**	3	12	8	10
**3**	3	14	10	10
**4**	4	15	10	10
**5**	4	20	15	10
**6**	4	30	20	10
**7**	5	30	20	20
**8**	5	35	25	20

**Table 7:** The general parameters for different scenarios.

Item	Value
The number of operations	[1, 5]
Transport time between job shops	[8, 11]
Transport time between machines	[1, 4]
Unit processing energy consumption (UPEX)	[10, 20]
Unit idle energy consumption	$UPEX\cdot \left[\frac{1}{10},\frac{1}{6}\right]$
Unit transport energy consumption	2
Due date tightness (DDT)	[0.5, 1.5]
Weight of job	[1, 5]

**Table 8:** The hyperparameters.

Hyperparameters	Values
Number of episodes	2000
Batch size	256
Learning rate	1 × 10^−4^
The update frequency of target Q-network	200
Buffer capacity	1 × 10^5^
Alpha in PER	0.6
Beta in PER	0.4

**Table 9:** The classical scheduling rules.

Rule	Description
**Job Assignment Scheduling Rules**	
First in first out (FIFO)	The earlier the job arrives, the higher the processing priority. When the same operation is performed with the same machine, the first job to arrive is processed first.
Most operation number remaining (MOPNR)	The more operations remaining, the higher the processing priority. When processing the same operation with the same machine, the arriving jobs are processed first with the most remaining operations.
Least work remaining (LWKR)	When processing different jobs, the job with the shortest remaining average processing time is preferred.
Most work remaining (MWKR)	When processing different jobs, the job with the longest remaining average processing time is preferred.
**Machine Assignment Scheduling Rules**	
Shortest processing time (SPT)	If more than one machine can process $O_{ij}$, the machine with the shortest processing time is selected.
Earliest end time (EET)	If more than one machine can process $O_{ij}$, the machine with the earliest end time of the previous process is selected.

**Table 10:** Comparison results of classical scheduling rules and composite scheduling rules.

	FIFO + SPT		MOPNR+SPT		LWKR+SPT		MWKR+SPT		Composite Scheduling Rules		Solution Distance (%)	
	TT ^1^	TE ^2^	TT	TE	TT	TE	TT	TE	TT	TE	TT	TE
Scenario 1	471	4226	1193	5480	549	4105	1277	5127	**19**	**3993**	0	0
Scenario 2	1141	**5946**	4064	9309	865	6228	3740	9782	**164**	6203	0	4.32
Scenario 3	1463	7622	1628	7922	1533	**7522**	1600	8247	**186**	8045	0	6.95
Scenario 4	1792	8357	3941	13,468	4242	**8001**	4836	11,829	**745**	8054	0	0.66
Scenario 5	3353	**9921**	2186	10,169	2608	10,279	3170	11,088	**942**	10,801	0	8.87
Scenario 6	4840	13,391	6901	16,760	7391	**12,554**	7876	16,930	**1136**	13,560	0	8.01
Scenario 7	2848	**12,368**	5539	17,424	4915	13,606	6103	17,893	**521**	14,149	0	14.4
Scenario 8	9522	**18,303**	**3953**	19,596	7234	20,961	5872	20,228	4896	21,850	23.85	19.37

^1^ TT represents the total weighted tardiness time of an algorithm in the table. ^2^ TE represents the total energy consumption of an algorithm in the table.

**Table 11:** Comparison results of classical scheduling rules, composite scheduling rules, D3QN with PER, and Rainbow DQN.

	Classical Scheduling Rule		Composite Scheduling Rule		D3QN with PER		Rainbow DQN		Solution Distance (%)	
	TT	TE	TT	TE	TT	TE	TT	TE	TT	TE
Scenario 1	471	4226	19	3993	58	7624	**16**	4106	0	2.82
Scenario 2	865	6228	164	6203	179	7843	**45**	**5917**	0	0
Scenario 3	1463	7622	186	8045	630	9521	**101**	**7620**	0	0
Scenario 4	1792	8357	745	8054	712	10,895	**391**	**7966**	0	0
Scenario 5	2186	**10,169**	942	10,801	986	18,080	**444**	10,961	0	7.78
Scenario 6	4840	13,391	1136	13,560	1104	13,962	**503**	**11,575**	0	0
Scenario 7	2848	**12,368**	521	14,149	1748	15,833	**212**	12,428	0	0.48
Scenario 8	3953	**19,596**	4896	21,850	3508	25,204	**2179**	22,191	0	13.24

## Data Availability

Enquiries about data availability should be directed to the first authors.
